# Multidisciplinary Approach for Bone Metastasis: A Review

**DOI:** 10.3390/cancers10060156

**Published:** 2018-05-24

**Authors:** Takahiro Kimura

**Affiliations:** Department of Urology, Jikei University School of Medicine, 3-25-8, Nishi-Shimbashi, Minato-ku, Tokyo 105-8461, Japan; tkimura@jikei.ac.jp; Tel.: +81-3-3433-1111; Fax: +81-3-3437-2389

**Keywords:** bone metastasis, metastatic cancer, bone management, cancer board

## Abstract

Progress in cancer treatment has improved the survival of patients with advanced-stage cancers. Consequently, the clinical courses of patients are prolonged and often accompanied by morbidity due to bone metastases. Skeletal-related events (SREs), such as pathological fractures and spinal paralysis, cause impairment in activities of daily life and quality of life (QOL). To avoid serious SREs causing impairment in QOL and survival, early diagnosis and a prophylactic approach are required. It is necessary to initiate a bone management program concurrently with the initiation of cancer treatment to prevent complications of bone metastasis. In addition, the requirement of a multidisciplinary approach through a cancer board focusing on the management of bone metastases and involving a team of specialists in oncology, palliative care, radiotherapy, orthopedics, nuclear medicine, radiology, and physiatrists has been emphasized. In the cancer board, a strong focus is placed on the prevention of complications due to bone metastases and on reductions in the high morbidity, hospitalization rate, and overall costs associated with advanced-stage cancers. Recent reports suggest the usefulness of such approaches. The multidisciplinary approach through a cancer board would improve QOL and prognosis of patients, leading to new or continued systemic therapy for primary cancers.

## 1. Introduction

The bone is one of the most common metastatic sites of cancers and bone metastases are a major cause of morbidity in the patients with advanced-stage malignant diseases [1]. Recent progression in cancer treatments, such as the development of molecular-targeted agents and immune checkpoint inhibitors, has improved the survival of patients with advanced-stage cancers [2]. Consequently, the clinical courses of patients are prolonged and often accompanied by morbidity due to bone metastases. Morbidities, such as pathological fractures and spinal paralysis, cause impairment in activities of daily life (ADLs) and quality of life (QOL), and affect prognosis because of deterioration of the affected patient’s general condition and discontinuation of treatment for the primary disease [3,4,5,6,7,8].

Formerly, the management of bone metastasis did not hold much importance because it had been considered palliative and unassociated with prognosis. Thus, bone metastases were sometimes diagnosed after developing serious complications, such as pain, pathological fractures, and spinal cord compression. Recently, it has been necessary to initiate bone management programs concurrently with the initiation of cancer treatment to effectively prevent serious complications of bone metastasis. In addition, the requirement of a multidisciplinary approach involving a team of specialists in oncology, palliative care, radiotherapy, orthopedics, nuclear medicine, radiology, and physiatrics has been emphasized [1]. This review describes recent developments in the multidisciplinary team approach for bone management of patients with cancer with bone metastasis.

## 2. Epidemiology of Bone Metastasis

Bone is a common metastatic site in patients with breast, lung, and prostate cancer [9,10,11,12,13,14], whereas it can also occur in other cancers, such as liver cancer, kidney cancer, and multiple myeloma [15]. In prostate cancer, bone is the most frequent metastatic site and more than 90% of patients develop bone metastasis 2 years prior to death [14]. The bone is also the leading metastatic site in breast cancer, both at initial presentation of metastases and as the site of first recurrence [16]. The bone is the third most common site of metastasis after the lungs and the liver [13,17]. On the other hand, bone metastases are the most frequent malignancy of the bone [13]. Bone metastasis typically occurs via hematogenous dissemination [18]. The most frequent sites of metastases in the bone are the lumbar vertebrae, followed by the thoracic vertebrae, cervical vertebrae, and sacrum, whereas metastases in the appendicular skeleton are rare [19]. Pathological fractures and spinal paralysis caused by metastases in the vertebrae or femur have serious influences on the ADLs and QOL of affected patients.

## 3. Pathophysiology of Bone Metastasis

The reason why bone is a favorite site of metastasis from many types of cancer is not yet fully understood. One possible reason is that the microenvironment of the bone marrow is appropriate for the growth of cancer cells. In the bone marrow, metastatic cancer cells take advantage of the normal marrow physiology to survive distant from the primary site [20,21]. The bone marrow houses both cells of hematopoietic lineage and cells responsible for bone remodeling, such as osteoblasts and osteoclasts. Metastatic cancer cells in the bone interact with osteoclasts or the hematopoietic stem cell niche, which play important roles in the early colonization of bone [22]. Disseminated tumor cells (DTCs) positively influence bone remodeling to create a favorable environment for further recruitment and better survival of DTCs in the bone marrow [21,23]. Local production of osteolytic factors by the DTCs stimulates osteoclast-mediated bone resorption, which induces the production of growth factors and secretion of osteolytic cytokines [15]. Bone resorption may also play a role in the formation of osteoblastic lesions in patients with prostate cancers [15].

Bone metastases can be lytic, blastic, or mixed depending on the type of cancer [1]. Osteoblastic metastases are typical in prostate cancer, and are sometimes detected in breast and undifferentiated type stomach cancer. Meanwhile, osteolytic metastases are detected in many types of cancers, such as breast, lung, thyroid, and stomach cancers. The frequency of serious complications depends on the site and type of lesion.

In addition, systemic treatment for patients with bone metastasis, such as hormone therapy, chemotherapy, and steroids, often influence the microenvironment of the bone and sometimes cause secondary osteoporosis. Aromatase inhibitors for breast cancer increase the risk of secondary osteoporosis and pathological fractures [24,25]. Androgen deprivation therapy (ADT) is a standard treatment for metastatic prostate cancer. Because the efficacy of ADT is very high and progression of prostate cancer is relatively slow, patients with prostate cancer are usually treated using ADT for long periods. Long-term administration of ADT yet increases the risk of secondary osteoporosis and pathological fractures, especially in elderly patients [26,27]. A prospective study evaluating bone mineral density (BMD) in a total of 84 patients with prostate cancer after administering ADT for 2 years revealed that BMD significantly decreased by 6.16% after 2 years compared to the baseline [27].

## 4. Skeletal-Related Events

Skeletal-related events (SREs) are skeletal complications associated with bone metastases, including cancer-induced bone pain, hypercalcemia, pathological bone fractures, and spinal cord compression [21]. Pain is one of the most frequent SREs in patients with cancer with metastatic disease; 68% of patients experience pain [28]. Meanwhile, other serious SREs, such as pathological fractures, spinal cord compression, and hypercalcemia, worsen patients’ QOL and reduce survival rates [3,4,5,29,30]. A retrospective cohort study including 832 patients with castration-resistant prostate cancer and bone metastasis revealed that 207 of them developed symptomatic skeletal events (SSEs) during follow-up period (median 2.1 years). Among them, 103 patients completed Functional Assessment of Cancer Therapy-Prostate (FACT-P) and Brief Pain Inventory-Short Form (BPI-SF) questionnaires. The SSE cohort had lower mean FACT-P functional well-being, higher mean pain severity and worst pain scores compared with the non-SSE cohort [29]. In addition, Howard et al. conducted a retrospective study including 233 men diagnosed with non-metastatic castration-resistant prostate cancer. During follow-up period (median 14.7 months), 88 (38%) patients had an SRE and 198 (85%) died. The SRE was significantly associated with increased mortality [30].

Information on the management of SREs is shown in Table 1. Importantly, randomized trials showed the effects of bisphosphonate and denosumab to prevent SREs in metastatic cancer. Zoledronic acid is the first bisphosphonate to demonstrate efficacy to reduce skeletal complications in patients with bone metastases from solid tumors including breast cancer, non-small cell lung carcinoma and multiple myeloma [31,32]. In addition, randomized trials showed the superiority of denosumab to zoledronic acid for prevention of SREs [33,34]. In the phase 3 randomized, double-blinded study, a total of 1904 patients with bone metastases from castration-resistant prostate cancer were assigned to either denosumab or zoledronic acid with a 1:1 allocation [34]. Median time to first SRE was significantly longer with denosumab than those with zoledronic acid (20.7 versus 17.1 months, respectively).

Nine to 29% of patients with bone metastases develop pathological fractures [35,36]. Pathological fractures not only reduce QOL, but also impair the survival of patients [37,38]. Pathological fractures are mainly treated with surgery to stabilize the fractured bones to improve QOL via pain relief and restoration of function and mobility [39]. Radiation therapy is administered only for supportive therapy to prevent local recurrence by eliminating residual disease [40].

Spinal cord compression is an oncological emergency as it leads to reduced survival and QOL if accurate diagnosis is not made and treatment is not performed [41]. The spinal cord is damaged by compression or by vascular compromising due to tumor growth. The damage can be irreversible if the arterial flow to the spinal cord is disturbed [42,43,44]. The symptoms are pain; motor weakness; sensory deficits; gait disturbance; and urinary, bowel, and sexual dysfunction [41,45]. Immediate treatment is essential for spinal cord compression. Steroids are the first-line treatment to stabilize vascular membranes, and reduce inflammation and edema. Even though the efficacy of radiation is promising, surgery was shown to be more effective to relieve compression [46]. In a randomized, multi-institutional, non-blinded trial, patients with spinal cord compression caused by metastatic cancer were assigned to either surgery followed by radiotherapy (*n* = 50) or radiotherapy alone (*n* = 51). The primary endpoint was the ability to walk. Significantly more patients in the surgery group (42/50, 84%) than in the radiotherapy group (29/51, 57%) were able to walk after treatment. This trial show the significant role of surgery for the treatment of metastatic epidural spinal cord compression. Prompt decision-making is very important to reduce damage to the spinal cord. The optimum dose and treatment regimen of radiation for spinal cord compression is still controversial. The short course radiotherapy is preferable because the survival prognosis of most patients with metastatic epidural spinal cord compression is only a few months [47]. However, the high daily doses might be more toxic and less effective for the treatment of acute compression and prevention of recurrence. Only a randomized trial showed the non-inferiority of short course radiotherapy to the longer course [48]. In this trial, a total of 203 patients with metastatic epidural spinal cord compression and poor to intermediate expected survival were randomly assigned to either 4 Gy × 5 in 1 week (*n* = 101) or 3 Gy × 10 in 2 weeks (n = 102). The primary endpoint was overall response regarding motor deficits at 1 month after radiotherapy, defined as improvement or no further progression. The overall response rates regarding motor function were not significantly different, 87.2% after 4 Gy × 5 and 89.6% after 3 Gy × 10. However, both of the regimens was still non-standard short schedules. Further randomized trials is required to compare them with a standard, more protracted schedule.

Hypercalcemia is a paraneoplastic syndrome frequently observed in patients with breast cancer, lung cancer, and multiple myeloma [49]. Cancer-related hypercalcemia is mainly mediated by the activity of parathyroid hormone-related proteins produced by cancer cells, followed by bone resorption due to bone metastases. Common symptoms of hypercalcemia are nausea, vomiting, anorexia, and abdominal pain. It can also cause cognition disorder or arrhythmia [49]. As treatment for hypercalcemia, hydration and diuretics are administered to promote renal calciuresis, and bisphosphonates and denosumab are administered to inhibit pathological bone resorption [49,50,51].

## 5. Early Diagnosis and Prophylactic Approach for Bone Management

Early diagnosis and a prophylactic approach for serious SREs, such as pathological fractures and spinal cord compression are required because they will cause impairment of QOL and survival once they occur. Bone management in the former clinical system used to be fragmentary and unsatisfactory in that patients were referred to a series of specialists, often with long waiting lists, which created great psychological stress and led to poor continuity of healthcare [1]. In addition, physicians often referred to orthopedic surgeons after the patients had developed fractures or paralysis. Although the therapy for bone metastases is usually palliative, early diagnosis and treatment are imperative to prevent irreversible neurological damage [52,53]. Unfortunately, these procedures are subject to “doctor’s delay” when the urgent nature of the clinical state is not apprehended [54,55].

There have been reports indicating that surgical intervention for patients with cancer with impending pathologic fractures lead better outcomes than that for patients with cancer with completed pathologic fractures [56,57]. In a retrospective study with a total of 182 consecutive patients who underwent surgery for metastatic disease of the femur, treatment of 97 impending pathologic fracture yielded better results than treatment of 85 completed pathological fractures with less average blood loss, shorter hospital stay, greater likelihood of discharge to home as opposed to an extended care facility, and greater likelihood of resuming support-free ambulation [57]. A population-based study also revealed that patients who underwent prophylactic stabilizations of bone metastatic disease had better survival outcomes than those who underwent surgical interventions after fracture [58]. In the study, a total of 624 patients who had undergone femoral stabilization, either for pathological femoral fractures or for prophylactic fixation of femoral metastases before pathological fractures were identified. Patients who underwent prophylactic stabilization had significantly better overall survival after adjusting for age, sex, comorbidities and type of cancer [58].

An understanding of the risk of pathological fractures in patients with bone metastases is an unmet need for prompt prevention, detection, and treatment. Mirels proposed a scoring system to quantify the risk of sustaining a pathologic fracture through a metastatic lesion in a long bone [59]. The scoring system was based on four characteristics: (1) site of the lesion; (2) nature of the lesion; (3) size of the lesion; and (4) pain. All the features were assigned progressive scores ranging from 1 to 3 (Table 2). This scoring system was extramurally validated and its reproducibility was demonstrated, in which the overall sensitivity was 91% and specificity was 35% [60,61]. A post-hoc analysis of prospective randomized trial of radiotherapy for patients with bone metastases from solid tumors also reported risk factors for pathological fractures with femoral metastases. Their study indicated that axial cortical involvement >30 mm and circumferential cortical involvement >50% were significant predictive factors of fractures, which were more reliable than Mirels’ scoring system [62].

In developing a care plan, the merits of prophylactic surgery should be considered for patients with bone metastases [15]. With early diagnosis, timely intervention is essential to prevent pathologic fractures.

## 6. Comprehensive Approach as a Cancer Board

**Figure 1:**
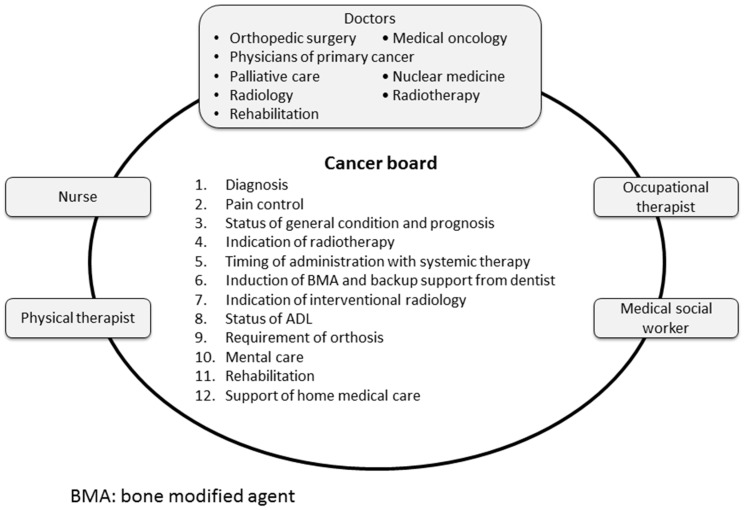
Cancer board focusing the management of bone metastases.

The treatment strategy for bone metastases from variable primary cancers should be planned comprehensively, taking into consideration the status of the primary cancer, prognosis, and social background of the patients. To achieve a multidisciplinary approach, a cancer board focusing on the management of bone metastases was organized involving a team of doctors in oncology, palliative care, radiotherapy, orthopedics, nuclear medicine, radiology, and rehabilitation; in addition, a nurse, physical therapist, occupational therapist, and medical social worker were included (Figure 1). 

A strong focus is placed on the prevention of complications due to bone metastases and on a reduction in the high morbidity, hospitalization rate, and overall costs associated with management of advanced-stage cancers [1].

Interdisciplinary meetings are held on a regular basis, every two to four weeks. An orthopedic surgeon or a medical oncologist usually coordinates members of the cancer board. In the cancer board of our institution, physicians of primary cancer or radiologists select cases and the radiologist presents an interpretation of the images. Issues that are focused on in the meetings are as follows: (1) confirmation of diagnosis of bone metastasis, (2) pain control by analgesic drugs or nerve-blocking agents, (3) status of general condition and prognosis, (4) indication of radiotherapy, (5) timing of administration with systemic therapy, (6) induction of bone-modifying agents and backup support from experts in oral surgery and preventive dentistry, (7) indication of interventional radiology, (8) status of ADLs, (9) requirement of orthosis, (10) mental care, (11) rehabilitation, and (12) support of home-based medical care [63]. In particular, clinical decisions to perform surgical interventions should be made in the context of the overall health status of the patients [15]. The major consideration is the functional status of the patient and what can be performed to preserve function from the point of view of bone metastases. Importantly, as surgical intervention requires a term of rehabilitation to restore function, patient prognosis is required to be predicted accurately.

In our institution, the cancer board is held monthly. Radiologists review all imaging studies of patients with advanced cancer and pick up cases those possibly require a prophylactic approach to prevent serious SREs in advance. This process is very important and effective in our experience. Physicians sometimes overlook signs of serious SREs such as pain, motor dysfunction and sensory disturbance if they are not sever or the patients have more serious symptom. The physicians review the medical records of the patient and the prophylactic approach is discussed multidisciplinarily. Physicians of primary cancer also pick up and present the patients those may require intervention.

Even though it is difficult to evaluate the efficacy of the multidisciplinary approach of cancer boards accurately, there have been reports suggesting the usefulness of such approaches. Ibrahim et al. reported preliminary results of a new organized Osteo-Oncology Center involving 19 specialists in their institution [1]. A total of 425 patients were assessed by the team in its first three years. Anonymous questionnaires about the quality of the service revealed that 98% of patients were very satisfied or satisfied, suggesting the high efficacy of the cancer board, especially in terms of decreasing psychological suffering. The management of bone metastases by such a multidisciplinary approach would improve the total health situation of patients, leading to new or continued systemic therapy for primary cancers. Further studies are required to evaluate the influence of cancer board on the oncological outcomes of patients with bone metastasis.

## 7. Current Pitfalls and Future Directions

Although the multidisciplinary approach of cancer boards is effective method for management of bone metastases, there are some pitfalls. It is sometimes difficult to manage patients impending to develop serious SREs timely by the meeting held biweekly or monthly. More instantaneous meeting using web conference systems might resolve this problem. Picking up patients those require such an approach is sometimes difficult. Therefore, the role of radiologists to pick up patients those have serious bone metastases is important. In addition, physicians should learn the method to diagnose signs of impending SREs by orthopedists. Accurate prediction of prognosis of the patient is also important. The prognosis of the patients influences on the approach to the SREs. However, physicians tend to predict the prognosis more optimistically. Such a multidisciplinary approach can establish common understanding among the member of the cancer board.

## 8. Conclusions

Bone metastases cause serious morbidities, such as pathological fractures and spinal paralysis, which cause decreases in ADLs and QOL. Treatment strategies for bone metastases should be planned comprehensively from several points of view of the health status, prognosis, and social background of the patients. Multidisciplinary approaches through a cancer board focusing on the management of bone metastases consisting of specialists will support comprehensive healthcare and treatment of patients.

## Figures and Tables

**Table 1:** Management of skeletal related events.

Skeletal Related Event	Management	Effects
Bone pain	NSAIDs, Opioids	Analgesic effects
	Bisphosphonates	Inhibition of pathological bone resorption Analgesic effects
	Denosumab	Inhibition of pathological bone resorption Analgesic effects
	Radiation	Analgesic effects Tumor shrinkage
Pathological bone fracture	Surgery	Stabilization of fracture
	Radiation	Supportive therapy to prevent local recurrence
	Bisphosphonates	Prophylaxis
	Denosumab	Prophylaxis
Spinal cord compression	Steroids	Stabilization of vascular membranes Reduction of inflammation and edema
	Radiation	Tumor shrinkage effects
	Surgery	Relief for the compression
	Bisphosphonates	Prophylaxis
	Denosumab	Prophylaxis
Hypercalcemia	Hydration	Promotion of renal calciuresis
	Loop diuretics	Promotion of renal calciuresis
	Bisphosphonates	Inhibition of pathological bone resorption
	Denosumab	Inhibition of pathological bone resorption

NSAID: nonsteroidal anti-inflammatory drug.

**Table 2:** Mirels’ scoring system for diagnosing impending pathologic fractures in a long bone [59].

Score	Site of Lesion	Size of Lesion	Nature of Lesion	Pain
1	Upper limb	<1/3 of cortex	Blastic	Mild
2	Lower limb	1/3–2/3 of cortex	Mixed	Moderate
3	Trochanteric region	>2/3 of cortex	Lytic	Functional
